# Identification of a Papain-like Cysteine Protease Functioning as an Avirulence Factor in Striga–Cowpea Interactions

**DOI:** 10.3390/plants14101427

**Published:** 2025-05-09

**Authors:** Danhua Zhang, Michael P. Timko

**Affiliations:** Department of Biology, University of Virginia, Charlottesville, VA 22904, USA

**Keywords:** avirulence, cowpea, cysteine protease, innate immunity, *Striga gesnerioides*

## Abstract

While most cowpea cultivars are susceptible to parasitism by the root parasitic weed *Striga gesnerioides* (Willd.) Vatke, cultivar B301 is resistant to all *Striga* races except for SG4z. Resistance to *Striga* parasitism is manifested by the elicitation of a hypersensitive response (HR) at the site of parasite attachment on the host root followed by rapid death of the attached parasite. We isolated a papain-like cysteine protease (PLCP) designated SGCP1 that is highly expressed in the haustoria of *S. gesnerioides* race SG3 at the time of parasite attachment to the host root. SGCP1 contains an apoplast-targeting signal peptide, a Cathepsin pro-peptide inhibitory domain, a papain family cysteine protease domain, and a granulin domain. Full-length SGCP1 and a variant lacking the signal peptide (SGCP∆SP) were expressed in the roots of composite B301 plants. Expression of SGCP1 and SGCP∆SP resulted in activation of host innate immune responses exemplified by increased frequency of HR and decreased levels of parasite cotyledon expansion (CE), indicative of successful host parasitism, in transgenic compared to wild-type B301 roots parasitized by SG4z. These data indicate that SGCP1 functions as an avirulence factor capable of activating host innate immunity and furthers our understanding of how compatible and incompatible host–parasite interactions are controlled.

## 1. Introduction

*Striga* species (witchweeds) are obligate root hemi-parasites in the Orobanchaceae family that attack staple food and forage crops throughout sub-Saharan Africa and the Indian subcontinent [1,2,3]. *S. gesnerioides* (Willd.) Vatke is one of the major forms of the parasite and primarily targets cowpea (*Vigna unguiculata* (L.) Walp.), an important grain legume in the semiarid Sahel regions of Africa, crucial for the livelihood and nutrition of countless millions of small-scale low-input farmers [4,5]. While most cowpea cultivars are susceptible to parasitism by *S. gesnerioides*, several resistant landraces and local accessions have been identified [5,6,7,8]. Resistance in these cowpea genotypes takes on one of two different phenotypic manifestations: a rapid localized cell death characteristic of a hypersensitive response (HR) at the site of parasite attachment to the host root with a subsequent discoloration and death of the parasite within 3–4 days, or little to no host root response at the attachment site but subsequent arrested growth of the parasite seedling early in the tubercle swelling stage of development [6,9,10]. In the case of tubercle arrest, the parasite fails to make vascular connections with the host and shows no evidence of cotyledon expansion. Susceptibility is characterized by post-attachment expansion of the parasites cotyledons, indicative of successful attachment to the host’s vascular system and subsequent completion of the parasite’s lifecycle.

It is now well documented that some cowpea genotypes (cultivars and local accessions) can resist being parasitized by *S. gesnerioides* isolates collected from some geographical locations but not others [6,7,8,9]. This differential resistance response has led to the understanding that different races (or pathotypes) of *S. gesnerioides* exist across West Africa. The cowpea cultivar B301 is resistant to almost all races of *S. gesnerioides* identified thus far and when parasitized by these races (e.g., SG1, SG2, SG3, SG5) develops an HR at the site of parasite attachment. Apoptosis of cells at the site of attachment, development of an HR and failure to make connections with the host vascular system leads to parasite death in these interactions [11]. In contrast, when race SG4z attaches to the root of B301, the innate immune response of the host is not activated, an HR does not occur, and the parasite successfully penetrates the host root cortex, expands its cotyledons, grows and completes its lifecycle [8].

Previous studies have shown that resistance to *S. gesnerioides* parasitism in cowpea is conferred by single dominant genes operating in a race-specific manner [12,13,14]. Using a molecular marker-assisted positional cloning strategy, Li and Timko [13] identified and cloned a gene from cowpea that confers resistance to race SG3 and showed that it encodes canonical resistance (R) protein with a N-terminal coiled-coil domain (CC), followed by a central nucleotide binding site (NBS) and a C-terminal leucine-rich repeat (LRR) domain. This gene, designated RSG3-301, is a critical component of effector-triggered immunity (ETI) in cowpea [13,15]. Subsequent studies demonstrated that the RSG3-301 R protein triggered HR elicited by race SG3 operates through a VuPOB1-mediated signal transduction pathway leading to differential gene expression associated with the defense response [16,17,18]. Overexpressing VuPOB1 in B301 roots resulted in a higher frequency of HR occurrence and a concomitant decrease in parasitism by SG4z, suggesting that VuPOB1 functions as a positive regulator of HR by activating host innate immunity gene expression in cowpea [18].

Comparative transcriptome analysis [17,18] was carried out to identify transcripts differentially expressed in the haustorial secretomes of *S. gesnerioides* SG3, SG4 and SG4z seedlings parasitizing B301 cowpea roots. Using pairwise comparisons of differentially expressed transcripts, these studies identified 32 candidate gene products annotated as possible virulence factors. In similar studies designed to identify components of *Striga* virulence, Qui et al. [19] and Bradley et al. [20] performed comparative transcriptomic analyses of the haustorial secretomes of *S. hermonthica*, *S. asiatica*, and two *Cuscuta* species and were able to identify candidate avirulence factors with varied proposed functions including cell wall modification and immune suppression, as well as protease, kinase, or peroxidase activities. Thus, it appears that *S. gesnerioides*, as well as other related *Striga* and *Cuscuta* species, likely possess in their haustorial secretones multiple avirulence factors capable of eliciting host resistance responses.

As noted previously, SG4z is a recently evolved race that does not elicit an HR on B301 and successfully parasitizes this host [8]. The virulence of SG4z could be attributed to either loss or diminution of specific virulence factors, the acquisition of novel effectors that allow the parasite to circumvent or suppress activation of the innate immunity in B301, or both. Su et al. [18] addressed this question by comparing the haustorial secretomes of SG3, SG4, and SG4z and were able to identify a gene specifically expressed in the SG4z secretome. This gene, dubbed *SHR4z,* encodes an effector protein secreted from the haustoria of SG4z, which specifically suppresses HRs in B301 under SG3/SG4 parasitism. In effect, SHR4z blocks the ability of avirulence factors present in the SG3 and SG4 haustorial secretomes that are recognized by the innate immune system of B301. Su et al. [18] showed that SHR4z targets VuPOB1, specifically blocking activation of innate immunity and elicitation of the HR conferred by RSG3-301 in B301, rendering it susceptible to SG4z parasitism.

It has been shown that papain-like cysteine proteases (PLCPs) are involved in a wide array of essential functions during plant growth, development, and defense [21,22,23,24]. Their role in plant defense extends beyond mere degradation of invasive proteins, as they participate in the modulation of defense-related signaling pathways, contributing to the orchestration of a robust immune response [21,25]. For example, the PLCP cathepsin-B is required for elicitation of an HR in *Nicotiana benthamiana* [26] as evidenced by the observation that silencing of cathepsin B prevents programmed cell death (PCD) and compromises disease resistance induced by *Erwinia amylovora* and *Pseudomonas syringae* pv. tomato. Silencing also suppressed the HR in *N. benthamiana* triggered by the potato R3a and *Phytophthora infestans* Avr3a avirulence factors [26]. Furthermore, silencing of the PLCP C14 ortholog in *N. benthamiana* increased its susceptibility to the oomycete pathogen *Phytophthora infestans* [27]. In *Arabidopsis thaliana*, the vacuolar PLCP ‘Responsive to Dehydration 21A’ (RD21A) has been shown to function in the plant defense response, with *rd21* mutant lines showing increased susceptibility to the necrotrophic pathogen *Botrytis cinerea* [23]. Transgenic rice lines, in which the *OCP* (oryzain alpha chain precursor) ortholog of *RD21* was knocked out, exhibit enhanced resistance against the blast fungus *Magnaporthe oryzae* [28].

Many pathogens produce effectors that target PLCPs to manipulate plant immunity [29]. For instance, the PopP2 effector from the bacterial pathogen *Ralstonia solanacearum* engages with RD19, leading to the relocation of the protease from vesicles into the nucleus [30]. The effector 4E02 of the sugar beet cyst nematode *Heterodera schachtii* targets RD21A and translocates it from the vacuole to the nucleus and cytoplasm, presumably hindering the protease’s ability to execute its defensive role [31]. The clubroot pathogen *Plasmodiophora brassicae* secretes effector SSPbP53 that can directly interact with and inhibit cruciferous PLCPs, specifically Arabidopsis XYLEM CYSTEINE PEPTIDASE 1 (AtXCP1) [32].

Some bacterial pathogens can produce PLCPs to manipulate defense responses in the plant cytoplasm. For example, HopPtoN is a cysteine protease effector from *Pseudomonas syringae* pv. tomato DC3000 that functions inside the plant cell to suppress *Pseudomonas*-induced necrosis [33]. Comparative microarray analysis identified a cysteine protease termed cuscutain specifically upregulated in haustoria of *Cuscuta reflexa* during host infection [34]. Application of the purified protease to leaf surface prior to infection was found to enhance host defense responses. Subsequently, Amini et al. [35,36] showed that the expression of inhibitory propeptide of cuscutain in transgenic plants effectively interrupted cuscutain enzyme activity and subsequently haustoria development at the endophytic stage making the host effectively resistant.

In the current study, we identified a PLCP that is differentially expressed in the haustorium of *S. gesnerioides* SG3 during its parasitism of its cowpea host. We identified the gene encoding this PLCP in the *S. gesnerioides* genome, designated *SGCP1*, and characterized its encoded protein. We demonstrate here that transgenic expression of the full-length SGCP1 cDNA or a truncated version encoding a SGCP1 protein without the signal peptide (SGCP∆SP) primes host innate immune responses in the roots of B301 allowing the host to resist parasitism by *Striga* race SG4z to which it is otherwise susceptible. The identification of an avirulence (Avr) factor in parasitic plants furthers our understanding of how compatible and incompatible host–parasite interactions are controlled, and such knowledge could eventually provide a potential strategy for controlling or mitigating the impact of parasitic plants on crop yields.

## 2. Results

### 2.1. Characterization of a PLCP from Striga Haustoria

The comparative transcriptome analysis pipeline was developed and used to identify transcripts differentially expressed in the haustorial secretomes of three races of *S. gesnerioides* (SG3, SG4 and SG4z), including two that formed incompatible interactions with B301 (SG3, SG4) and one (SG4z) that formed a compatible [17,18]. Pairwise comparisons of the haustorial secretomes of SG3, SG4 and SG4z identified 32 contigs not only differentially expressed between incompatible and compatible races but differentially expressed in SG3 relative to SG4 and SG4z, and whose encoded proteins were annotated as possible virulence factors. One contig SGall_097996.2 was of particular interest since it encoded putative papain-like cysteine proteases (PLCPs) and CP superfamily members have been previously identified as virulence factors in other plant–plant pathogen interactions [24,37,38].

To obtain the full-length coding sequences of contig SGall_097996.2, we used Standalone BLASTN to identify the gene coding region in the SG3 genome assembly. We subsequently designed gene-specific forward and reverse primers and then used PCR to amplify a full-length cDNA from SG3 haustorial cDNA. We designated this gene coding region as *SGCP1*.

**Figure 1:**
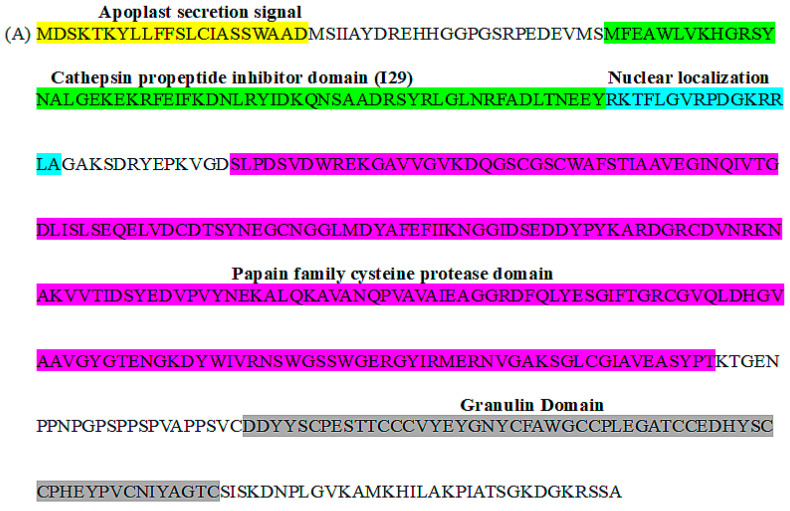

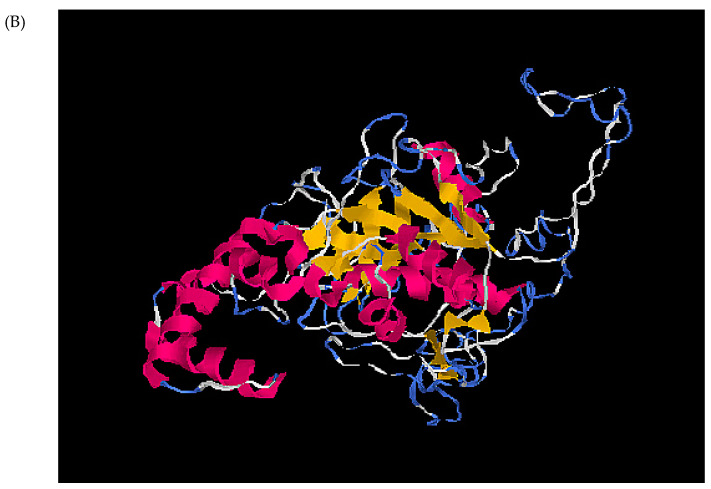
Domain structure of SGCP1 and predicted protein structure. (**A**) The predicted protein structure of SGCP1 along with its identified functional domains. (**B**) The result of modeling the predicted protein structure of SGCP1 using the I-TASSER [39]. The figure depicts the highest ranked model (with a confidence score for estimating the quality of predicted model (C-score = −2.56) and estimated measures of the predicted model to obtain structures of TM-score = 0.42 ± 0.14; and RMSD = 13.4 ± 4.1Å. In the figure blue indicates an alpha-helix, red indicates a beta-sheet, and yellow represents a coil or irregular secondary structure.

The *SGCP1* coding region is 1395 nucleotides (nt) in length and encodes a 464-amino acid (aa) protein containing a predicted 20-aa extracellular (apoplast)-targeting signal peptide and 57-aa Cathepsin pro-peptide inhibitory domain at the N-terminus, a 216-aa papain family cysteine protease domain centrally, and a 57-aa granulin domain at the c-terminal end. Based upon protein modeling (Figure 1B), the cysteine protease domain in SGCP1 contains a catalytic triad formed by the amino acids 160-Cys, 296-His and 316-Asn. This is consistent with the fact that cysteine proteases are hydrolase enzymes that share a common catalytic mechanism involving a nucleophilic cysteine thiol in a catalytic triad or dyad. Furthermore, SGCP1 also features a nuclear localization sequence (RKTFLGVRPDGKRRLA) between the inhibitory domain and the protease domain.

**Figure 2:**
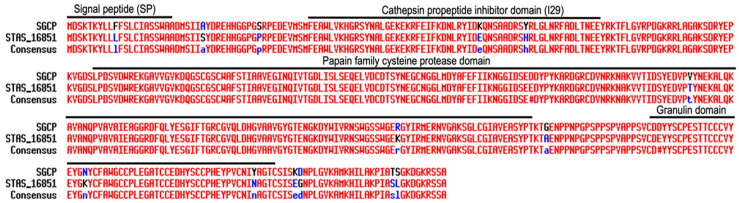
Protein sequence alignment of SGCP1 with its *S. asiatica* homolog. Alignment of SGCP1 with a cysteine protease homolog from *S. asiatica* (STAS_16851, GenBank: GER40192.1). The alignment was generated by MultAlin version 5.4.1 [41]. Red lettering indicates identical amino acids between the two homologous proteins (100% identity); black lettering indicates residues that are not identical; and blue lettering indicates conserved residues. The Signal Peptide (SP), Cathepsin propeptide inhibitor domain (129), papain family cysteine protease domain, and Granulin domain are indicated by black lines.

To determine if the SGCP1 protein was unique to *S. gesnerioides*, we surveyed available sequence information for other *Striga* species and found that it exhibits 96.6% similarity with a predicted probable cysteine protease from *S. asiatica* (STAS_16851, GenBank: GER40192.1) (Figure 2), a related witchweed that parasitizes members of the Poaceae [40]. Blast results against the Phytozome 13 database also indicate that it has high identity to Cathepsin L cysteine proteinases from *Lindenbergia philippensis* [Liphi.04G184200 (PAC:51277246)] (86% identity) and *Mimulus guttatus* var. IM767 [MgIM767.14G260500 (PAC:64867070)] (78% identity), two evolutionarily closely related but non-parasitic species [3].

### 2.2. Expression of SGCP1 and SGCP∆SP in B301 Roots Enhances HR and Suppresses Parasite CE When Challenged by SG4z

To gain insight into the possible role of the SGCP1 protein in parasite–host interactions, two plasmid constructs were created (pK7WG2D-SGCP-HA and pK7WG2D-SGCP∆SP-HA): one capable of expressing the full-length protein the SGCP1 protein (SGCP -HA) and the other lacking the apoplast targeting signal (SGCP∆SP-HA). We rationalized that the truncated form of the protein is likely the form being transferred to the host root from the surface of the haustorium. The two constructs were then used to generate composite B301 plants expressing the SGCP -HA and SGCP∆SP-HA proteins in their roots. The transgenic and non-transgenic roots on these B301 composite plants were then subjected to SG4z inoculation.

**Figure 3:**
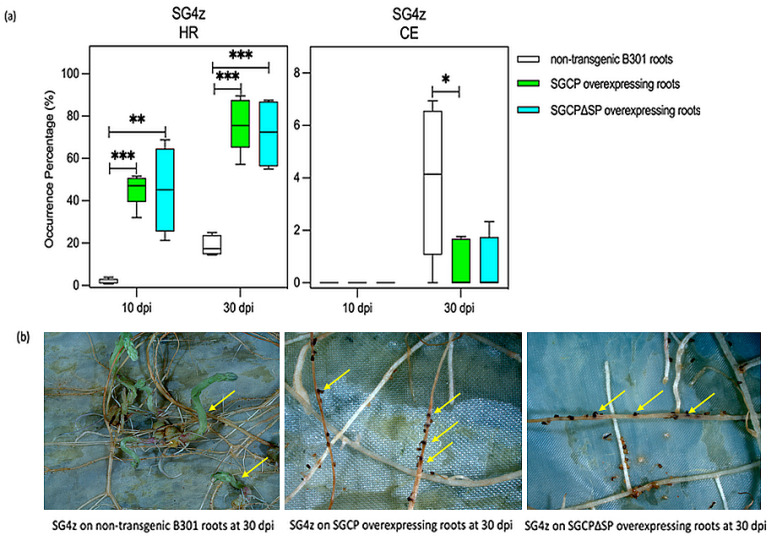
Expression of SGCP1 and SGCP∆SP in B301 roots enhances HR and suppresses parasite CE when challenged by SG4z. (**a**) Ex vitro composite B301 plants were generated that express the SGCP and SGCP∆SP proteins in their roots. Transgenic and non-transgenic B301 roots were inoculated with 2 d pre-germinated SG4z seedlings, and the phenotypic responses (HR, hypersensitive response; CE, cotyledon expansion) were scored at 10- and 30-days post-inoculation (dpi). The interaction event frequencies of HR and CE were obtained by counting the number of each event category and dividing by the total number of attachment events on transgenic and non-transgenic roots of each host plant. Statistical analysis was performed using the unpaired two-tailed *t* test on >4 independent host plant replicates (non-transgenic roots 10 and 30 dpi, *n* = 5; SGCP overexpressing roots 10 and 30 dpi, *n* = 5; SGCP∆SP overexpressing roots 10 and 30 dpi, *n* = 4). Boxplots indicate the median (horizontal lines), 25th and 75th percentile range (boxes) and minimum and maximum values (whiskers). A minimum of 25 events per root and at least 400 events per plant were scored. The asterisks indicate significant difference (* *p* < 0.05; ** *p* < 0.01; *** *p* < 0.001). (**b**) Representative photographs illustrating the phenotypic response of non-transgenic and transgenic B301 roots overexpressing SGCP and SGCP∆SP when parasitized by SG4z at 30 dpi. In the left panel, the arrows indicate the development of the SG4z parasite on non-transgenic B301 roots. The arrows on the middle and right panel highlight where the SG4z parasite has resulted in an HR and parasite death due to the presence of the SGCP transgene.

As shown in Figure 3a, the roots expressing SGCP-HA exhibited a significantly increased incidence of HR compared to the control non-transgenic B301 roots when challenged by SG4z, with significant differences observed at both 10 and 30 dpi (10 dpi *p*-value = 0.0002; 30 dpi *p*-value = 0.00001). Furthermore, these transgenic roots showed a significant reduction in cotyledon expansion (CE) events at 30 dpi (*p*-value = 0.04) (Figure 3a,b). Roots expressing SGCP∆SP-HA also demonstrated a significantly increased HR frequency compared to control non-transgenic roots at 10 and 30 dpi (10 dpi *p*-value = 0.002; 30 dpi *p*-value = 0.0002). There were no differences between B301 roots expressing SGCP-HA and SGCP∆SP-HA regarding HR and CE. As shown in Figure 3b, at 30 dpi, B301 roots expressing SGCP-HA or SGCP∆SP-HA demonstrated a strong inhibition effect in the cotyledon expansion of attached SG4z seedlings.

### 2.3. Molecular Characterization and Cellular Localization of Overexpressed SGCP1 in B301 Roots

**Figure 4:**
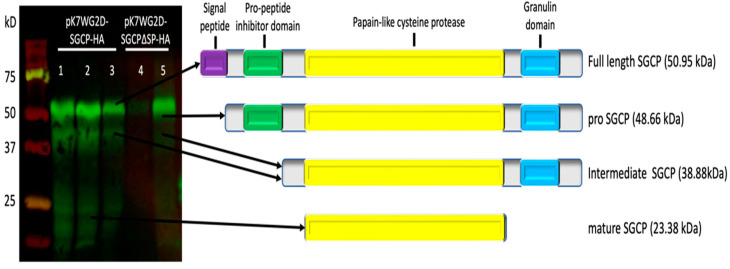
Immunoblot analysis of SGCP1-HA and SGCP∆SP-HA expressed in transgenic cowpea roots. The full-length SGCP1 proteins with HA epitope tag fused to the C-terminus and a truncated version lacking the signal peptide (SGCP∆SP-HA) were expressed in ex vitro composite B301 plants. Immunoblotting was carried out on total root protein extracts transgenic roots using anti-HA antibody. Lanes 1–3, roots expressing SGCP-HA; lanes 4–5, roots expressing SGCP∆SP-HA. The molecular weights SGCP-HA and SGCP∆SP-HA were predicted by the Compute pI/MW tool to be 50.95 kDa and 48.66 kDa, respectively. The lower molecular weight forms seen on the gel correspond to the processed intermediate and mature forms generated in vivo. Molecular weight markers are given on the left.

SGCP1 is predicted to be a pre-pro-protein, with an apoplast-targeting signal and an inhibitor propeptide that is removed intracellularly in the host when it is converted to an active mature form. Immunoblot analysis of total protein extracts from roots of B301 composite plants expressing *SGCP-HA* and *SGCP∆SP-HA* transgenes showed that both the SGCP1 and SGCP∆SP proteins were detected in the transgenic roots (Figure 4). As might be expected, the apoplast-targeting signal in SGCP appears to be removed by the host cell yielding a protein of similar size to the expressed truncated form. We also see evidence in the immunoblot analysis of immunoreactive lower molecular weight forms of the SGCP1-HA that are consistent with cellular processing and removal of the propeptide inhibitor domain and granulin domain, leading to the mature active form of the SGCP1-HA retaining only the cysteine protease domain. The fact that the majority of immunoreactive SGCP1-HA retains the propeptide inhibitor domain suggests that the SGCP1-HA would not be functional to assist in the SG4z parasitism activity.

**Figure 5:**
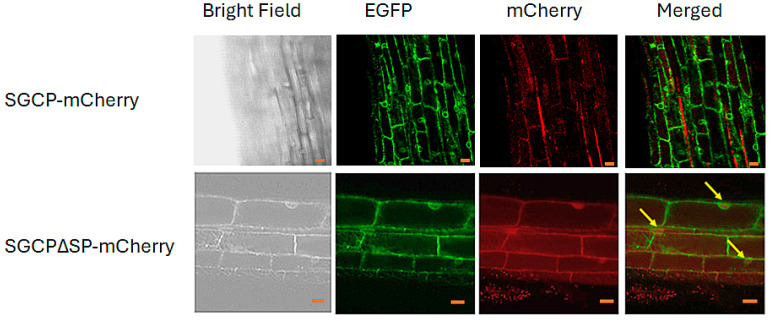
Subcellular localization of SGCP-mCherry and SGCP∆SP-mCherry fusion proteins. The figure shows representative photographs of the subcellular localization of SGCP-mCherry and SGCP∆SP-mCherry fusion proteins in transgenic B301 cowpea roots as viewed by confocal microscopy. B301 roots were transformed using *Agrobacterium rhizogenes* R1000 containing the pK7WG2D-SGCP-mcherry and pK7WG2D-SGCP∆SP-mcherry plasmids. The left panel is a brightfield image, the second from left is the image channel showing the EGFP marker for transformation, the mCherry lane shows the signal from the target transgene expression and the right panel is the Merged image (an overlay of the two). Visualization of mCherry was performed by excitation at 587 nm and monitoring emission at 610 nm (using a 564–630 nm range filter), and EGFP visualization was performed by excitation at 488 nm and monitoring emission at 509 nm (using a 497–544 range filter). Arrows indicate signal in nucleus. The images were captured at 100× magnification; bars, 10 µm.

To better understand the cellular localization of SGCP1 in the host cell, we created two transgene expression plasmids, pK7WG2D-SGCP-mCherry and pK7WG2D-SGCP∆SP-mCherry, one expressing the full-length SGCP1 protein fused with an mCherry reporter, and an analogous mCherry fusion protein lacking its apoplast targeting sequence. The two proteins, SGCP-mCherry and SGCP∆SP-mCherry, respectively, were expressed in B301 roots and the presence of the fusion proteins examined in the transgenic root tissue by confocal microscopy (Figure 5). While it was possible to detect both the SGCP-mCherry and SGCP∆SP-mCherry proteins by immunoblot analysis, we did not observe the full-length SGCP1-mCherry protein in cells. Rather, since the SGCP1 protein features an apoplast targeting signal, we detect the full-length SGCP1-mCherry protein outside the cells or near the plasmamembrane. This is consistent with the protein being targeted to the cowpea apoplast where it is subjected to rapid turnover. On the other hand, the truncated form of SGCP∆SP-mCherry protein does not accumulate in the cytoplasm to a large extent, although we did observe a faint nuclear localized signal in roots expressing the truncated protein, consistent with the observation that SGCP1 also features a nuclear localization sequence between the inhibitory domain and the protease domain.

## 3. Discussion

PLCPs play crucial roles in various biological processes in a wide range of organisms and in plants are integral to the regulation of endogenous processes, including development, senescence, hormone signaling, and immune responses [42,43]. In contrast to PLCPs in plants, those secreted by bacterial and fungal plant pathogens (e.g., Avr2, avrRpt2, YopT and AvrPphB) interfere with plant immunity. For example, AvrPphB from *Pseudomonas syringae* prevents the activation of RPM1-mediated resistance in *A. thaliana* [44]. Similarly, *P. syringae* AvrRpt2 degrades RIN4 and induces RPS2-mediated resistance [45]. The *Xanthomonas campestris* effector XopD significantly reduces the level of SUMO-protein conjugate host cells during infection of the fungus when it is heterologously expressed in plant cells [46]. XopD is a member of the ULP1 family of cysteine protease and while XopD host target substrates remain to be determined, there is a suggestion that they affect proteins with critical nuclear functions.

Similarly, two cysteine proteases secreted by *Phytophthora parasitica*, designated PpCys44 and PpCys45, act as virulence factors that trigger cell death in various *Nicotiana* spp. [47]. Expressing PpCys44 and PpCys45 with or without a signal peptide in *N. benthamiana* leaves resulted in different outcomes, with the protein containing signal peptide sequence inducing cell death in *N. benthamiana* leaves, whereas the protein without a signal peptide did not induce cell death. This may result from differences in cellular localization and ability to interact with cellular targets.

**Figure 6:**
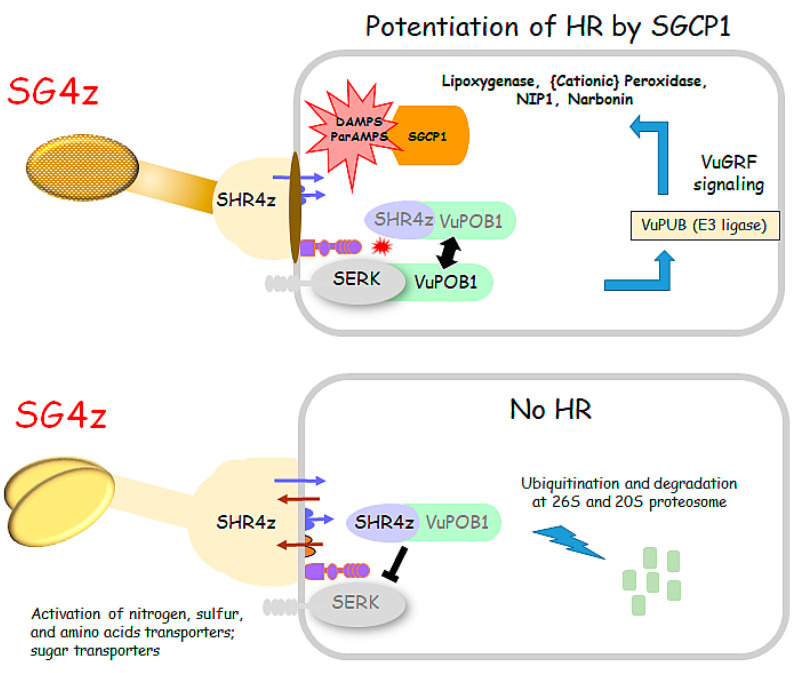
Model of compatible and incompatible interactions between *Striga* race SG4z and B301 with and without potentiation by transgenic SGCP1 and SGCP∆SP expression. The lower panel illustrates the current state of understanding of how SG4z overcomes host innate immunity by secretion of the suppressing effector SHR4z that leads to the proteolysis of VuPOB1 that is required for mounting an HR [18]. Expression of SGCP1 and SGCP∆SP leads to the formation of DAMPs that potentiate innate immunity, allowing the B301 root to shift the balance in favor of immunity versus susceptibility prior to SG4z attachment.

Here, we report the identification of a PLCP (SGCP1) that is expressed in the haustorium of the root parasitic plant *S. gesnerioides* at the time of host contact. Transgenic expression of SGCP1 or a truncated form of the protein, SGCP∆SP, resulted in the activation of defense responses in the roots of B301 composite plants, converting the normally SG4z susceptible cultivar to a resistant phenotype as evidenced by increased frequency of HR and inhibition of cotyledon expansion of attached SG4z seedlings (Figure 6).

The underlying mechanism by which SGCP1 enhances host resistance against *Striga* infection remains unclear and further studies are needed. Direct screening for interactive host proteins using yeast two-hybrid assays with SGCP1 cysteine protease domain as the bait and the B301 root cDNAs as the prey library failed to identify reproducible host interaction targets, even when the critical cysteine residue (C160) likely to be involved in the proteolysis was mutated to alanine. Nonetheless, we did observe that expression of SGCP1 and SGCP∆SP leads to a priming or potentiation of the innate immune response in transgenic B301 roots as evidenced by increased resistance to SG4z parasitism (e.g., significantly higher frequency of HR events) compared to non-transgenic B301 roots. In this case, the presence of SGCP1 allows the cells to jumpstart resistance prior to SG4z attack, leading to an ability to overcome either fully or partially the effect of the suppressing effector of HR SHR4z [18].

Defense priming has been shown to be an adaptive part of induced resistance [48,49,50]. In this regard, PLCPs may function indirectly by releasing damage-associated molecular patterns (DAMPs) from cellular components that are in turn recognized by plant receptors activating immune responses. Conversely, PLCPs may be serving directly as Avr factors that prime defense responses [51,52,53]. By analogy, although the nature of the encoded product is not known, it has been shown that the product of a dominant Avr gene in *Orobanche cumana* triggers Or5 resistance in sunflower [54].

Candidate virulence genes have been identified in *S. hermonthica* through a genotype–environment association study using redundancy analysis to test associations of 400,000 genic SNPs with virulence phenotypes across rice hosts [19]. The results found numerous candidates in the haustorial secretome (including pectin acetylesterases, glycosyl hydrolases and multi-copper oxidases) previously implicated in the growth and differentiation of haustoria [55]. Recently, Bradley et al. [20] conducted a comparative secretome analysis of *Striga* (*S. hermonthica* and *S. asiatica*) and *Cuscuta* (*C. campestris* and *C. australis*) species that identified 39 genes encoding putative virulence factors with proposed functions such as cell wall modification, immune suppression, protease, kinase, or peroxidase activities. Based on these studies, it appears that *Striga* species and likely related parasitic plant species contain suites of Avr factors that they employ to overcome host innate immunity. The identification of SGCP1 in the current study constitutes the first clear evidence that virulence factors predicted to exist in parasitic weeds can function directly to activate host innate immunity.

How SGCP1 interacts with the RSG3-301-mediated resistance pathway in the proposed gene-for-gene model for resistance in Striga–cowpea associations [13,18] remains to be determined in future studies. Such a role would be consistent with a multitiered interaction between the host and parasite and not unexpected since previously we have shown that *Striga* haustoria can release decoy effectors capable of repressing activation of the HR in host roots as a means for gaining ingress and completing their parasitic life cycle [18]. Such effectors would be needed to suppress the activation of HR resulting from the sensing of parasite virulence factors.

Most root parasitic weeds, fungi and nematodes form penetrating structures to gain entrance into host tissue and synthesize a cysteine protease activity essential to parasitism. Clearly, however, a duality exists in plant defense responses with respect to the role of PLCPs. On the one hand, they are required for full resistance of plants to various pathogens, whereas they are also targeted by secreted pathogen effectors to suppress immune responses. Cuscutain is a PLCP found in the haustoria of the arial parasite dodder (*Cuscuta reflexa*) that is essential for parasite ingress into the host plant likely by weakening host cellular structures through protein degradation [34]. Cuscutain is synthesized in the haustoria as pre-pro-protein, with an apoplast-targeting signal and an inhibitor propeptide that is removed intracellularly in the host, converting it to an active form. Bleischwitz et al. [34] showed that application of a solution of the custutain signal peptide-less propeptide to tobacco leaves acted as an inhibitor of parasite growth in *Cuscuta*–tobacco interactions without significantly affecting tobacco host development. Subsequently, Amini et al. [35] showed that transgenic tomatoes expressing the pre-propeptide part of cuscutain are less susceptible to *C. campestris* than untransformed wild-type plants and Amini et al. [36] reported that expression of the cuscutain propeptide in transgenic alfalfa plants effectively suppressed haustorial growth in *Cuscuta* at an early developmental stage, preventing the parasite from gaining ingress into the host leaf.

More recently, transgenic bioassays examining the role of the cysteine protease present in *Impomea batata* (ICP-1) carried out by Saify Nabiabad et al. [56] demonstrated that the ICP-1 propeptide can act as a defense molecule against plant parasites by inhibiting the potential function of parasite-cysteine proteases including those of *Globodera rostochiensis* (GCP-1), *Meloidogyne incognita* (MCP-1), *Heterodera glycines* (HCP-1), *Cuscuta chinesis* (CCP-1) and *Orobanche aegyptiaca* (OCP-1).

Based on our structural analysis of the SGCP1 protein, it contains both an apoplast-targeting prepeptide and a propeptide subunit (Figure 1) that folds on the active site and inhibits the function of the cysteine protease The fact that transgenic expression of SGCP1 or a truncated form of the protein, SGCP∆SP, results in decreased parasitism of B301 by SG4z is consistent with the above observations that expression of inhibitory propeptides can result in the blocking of parasite ingress. While there is some evidence in our immunoblot analysis for the conversion of SGCP1 and SGCP∆SP to lower molecular weight processed forms, the majority of the immunodetectable protein appears to be the full-length protein. Additional studies are needed to confirm whether expression of processed forms lacking the inhibitory propeptides that are also protective against SG4z.

The selection and breeding of resistant cultivars, accompanied by the use of modified agronomic practices, has provided some assistance to low-input farmers in sub-Saharan Africa dealing with the burden of Striga-infested soils. However, broadly effective control methods remain elusive especially because parasite evolution can outpace innate immunity as demonstrated in the case of the SG4z–B301 interaction [8]. Understanding the molecular mechanism of *Striga* virulence and the evolutionary changes in the parasite that contribute to breaking host resistance enhances our abilities to better solve this problem. The current work furthers our understanding of how compatible and incompatible host–parasite interactions are controlled. Thus, our findings here on the nature of Avr factors and their function could potentially contribute to the development of novel strategies for controlling *Striga* and other parasitic weeds and thereby enhancing plant productivity and food security worldwide.

## 4. Materials and Methods

### 4.1. Plant Materials

All experiments involving viable *S. gesnerioides* seeds, developing parasites, and host–parasite interactions were performed at the University of Virginia in an APHIS-approved Striga quarantine facility in the Department of Biology (Facility Number PPQ-VA-300069; APHIS Plant Protection and Quarantine Permit No. 526-23-279-17457). B301 seeds were kindly provided by Dr. Lucky Omoigui (Joseph Sarwuan Tarka University, Makurdi Nigeria). SG4z seeds were initially collected from plants growing in infested cowpea fields in Zakpota, Benin in 2009, and then grown on B301 plants through several generations in pots in the *Striga* Research Laboratory to ensure uniformity of virulence. For this purpose, SG4z seeds were blended with fine sand (<250 microns) to achieve a density of ~2000 seeds/gm. One gram of this sand–seed mixture was mixed into the top layer of a 20 cm diameter pot filled with autoclaved sand–soil mix [8] and the pots were irrigated every 2 d to preserve soil moisture and break *Striga* seed dormancy. After 7 d, four cowpea B301 seeds were sown in each pot and seedlings grown under a 12 h light/12 h dark cycle at 30 °C. After 6–8 weeks, parasite shoots emerged from the soil. Emerged *S. gesnerioides* SG4z seedlings were allowed to grow to maturity, self-fertilize and then the seeds were harvested. The time to maturity was about 3 months. The SG4z seeds were then dried at room temperature and stored until needed for inoculation.

### 4.2. Cloning of SGCP1 cDNA and Structural Analysis of SGCP1 Protein

Comparative transcriptomic was used to identify transcripts differentially expressed in the haustorial secretomes of three races of *S. gesnerioides*, including two that form incompatible interactions with B301 (SG3, SG4) and one (SG4z) that forms compatible interactions [17,18]. Pairwise comparisons were performed between the haustorial secretomes and contigs differentially expressed in SG3 relative to SG4 and SG4z were further examined. A total of 32 contigs were identified as possible virulence factors based on their apoplast-targeting signal. One contig SGall_097996.2, encoding putative papain-like cysteine proteases (PLCPs), was selected for additional study.

In order to identify the full-length coding region associated with contig SGall_097996.2, we used Standalone BLASTN [57] with its default parameters, with the exception of setting an e-value to 1e-7, to perform alignments against the SG3 genome assembly developed in collaboration with Professor Longjiang Fan (Zhejiang University, Hangzhou, China). The full-length gene coding region was identified using the FGENESH gene-finding tool (http://www.softberry.com/berry.phtml?topic=fgenesh&group=programs&subgroup=gfind, accessed on 6 July 2021) and was designated as *S. gesnerioides* cysteine protease 1(SGCP1).

To clone the full-length CDS of *SGCP1*, SG3 seedlings were grown on cowpea cultivar Blackeye and total RNA was extracted from haustorial tissue collected at each of three infection time points (0, 3, and 10 dpi). The total RNAs extracted from these samples were combined and 1 µg was then reverse transcribed into cDNA. RT-PCR reactions were performed using gene-specific forward and reverse primers (Forward: 5′-ATGGATTCTAAAACAAAATATCTGC-3′; Reverse: 5′-TCAAGCACTGCTCCTTTTCCCA-3′) designed against the predicted coding sequence of SGCP1. The amplified products were gel purified with Zymoclean Gel DNA Recovery Kit (Zymo Research, cat#D4008) and blunt-end cloned into pJET1.2 vectors (CloneJET PCR Cloning kit, ThermoFisher Scientific, Waltham, MA, USA, cat#K1232) for sequencing.

The molecular size of the protein was predicted by the Compute pI/Mw tool (https://web.expasy.org/compute_pi/, accessed on 22 April 2022). The signal peptide of the protein was identified by SignalP 6.0 (https://services.healthtech.dtu.dk/services/SignalP-6.0/, accessed on 22 April 2022). The transmembrane topology was predicted by DeepTMHMM (https://dtu.biolib.com/DeepTMHMM, accessed on 22 April 2022). The localization of the secreted proteins was determined by LOCALIZER (https://localizer.csiro.au/index.html, accessed on 22 April 2022.) and WoLF PSORT (https://www.genscript.com/wolf-psort.html, accessed on 22 April 2022). Protein domains were analyzed using HMMER software (https://www.ebi.ac.uk/Tools/hmmer/, accessed on 22 April 2022.) and NCBI Conserved Domain Search (https://www.ncbi.nlm.nih.gov/Structure/cdd/wrpsb.cgi, accessed on 22 April 2022.). Protein modeling was carried out using I-TASSER [39]; https://zhanggroup.org/I-TASSER/, accessed on 4 September 2024).

### 4.3. Construction of Transgene Overexpression Plasmids and Generation of Ex Vitro Composite Plants

An HA-epitope tag (YPYDVPDYA) was added to the C-terminus of SGCP1 coding regions by PCR amplification using gene-specific primers and the resulting product was then mobilized into the Gateway^®^ pDONR^™^221 Vector (Invitrogen, Carlsbad, CA, USA) using Gateway BP Clonase Enzyme mix (Invitrogen, Carlsbad, CA, USA). A truncated version lacking the predicted signal peptide (SGCP∆SP) with an HA-tag added to the C-terminus was also cloned into the Gateway^®^ pDONR^™^221 Vector. In construct SGCP∆SP-HA, an ATG start codon was inserted at the 5′ end of the truncated sequence for translation initiation. Both pDONR-SGCP-HA and pDONR-SGCP∆SP-HA were further recombined with the overexpression vector pK7WG2D [17] using Gateway LR Clonase Enzyme mix (Invitrogen, Carlsbad, CA, USA).

B301 seeds were surface sterilized with 10% (*v*/*v*) bleach for 10 min, washed 3 times (5 min each) in sterile water and grown in sterile rockwool. Ex vitro composite plants were generated as previously described [58] using *A. rhizogenes* strain R1000 carrying the pK7WG2D-SGCP-HA and pK7WG2D-SGCP∆SP-HA plasmids. Cowpea seedlings with regenerated roots were moved to rhizotrons containing granulated rockwool 20 d after transformation and grown at 30 °C for 14 d before inoculation with pre-germinated Striga SG4z seedlings. The veracity of transgene expression in the transgenic composite roots was confirmed by qRT-PCR using total RNA extracts from transgenic and non-transgenic tissues and gene specific primers as described above for cloning.

### 4.4. Striga–Host Root Interaction Assays

*S. gesnerioides* SG4z seeds were surface sterilized and germinated using host root exudate as previously described [17,18]. Germinated SG4z seedlings were gently transferred to transgenic and non-transgenic B301 roots with very fine point tweezers. Between 8 and 10 transgenic or non-transgenic roots were inoculated on 4 or more individual plants per treatment and the full experiment was replicated at least twice. Transgenic and non-transgenic roots of relatively equivalent age on the composite plants were inoculated with equivalent numbers of germinated *Striga* seedlings. To ensure a lack of bias, at 10- and 30-days post-inoculation (dpi), we scored only the successful parasite-host root interaction events (i.e., number of attachments) and then calculated the percentage for each interaction type (HR and CE) based on the total number of events. Across individual biological replicates (*n* = number of plants) a minimum of 25 events per root and at least 400 events per plant were scored. The data were combined from two replicate experiments and occurrence percentages for each interaction event were subject to unpaired *t* test for statistical analysis.

### 4.5. Immunoblot Analysis of SGCP Expression

Transgenic or non-transgenic roots (100 mg) were collected, flash frozen in liquid nitrogen and ground to fine powder. Ice-cold lysis buffer (10 mm Tris-HCl, pH 7.5, 150 mm NaCl, 0.5 mm EDTA, 0.5% (*v*/*v*) NP-40, 0.09% (*w*/*v*) Na-azide, protease inhibitor tablets (Sigma-Aldrich, St. Louis, MO, USA) and 1 mm PMSF) were added and the mixture further ground. The homogenate was centrifuged at 13,000 rpm at 4 °C. The supernatant was collected and suspended in 2 X SDS sample buffer (124.8 mm Tris-HCl, pH 7.5, 4% (*w*/*v*) SDS, 4% (*v*/*v*) beta-mercaptoethanol, 20% (*v*/*v*) glycerol, 0.02% (*w*/*v*) bromophenol blue) and de-natured by boiling at 95 °C for 10 min. The protein extracts were analyzed by SDS-PAGE and blotted to a PVDF membrane (Bio-Rad, Hercules, CA, USA, cat#1620177). The membrane was blocked by incubation in PBS-T buffer (10 mm Na2HPO4, 1.8 mm KH2PO4, pH 7.4 containing 137 mm NaCl, 2.7 mm KCl, 0.1% (*v*/*v*) Tween-20) with the addition of 5% (*w*/*v*) non-fat milk powder for 1 h. Next, the membrane was incubated with anti-HA antibody as the primary antibody (ThermoFisher Scientific, cat#26183, diluted 1:1000) at room temperature for 1 h and then washed 3 times with PBS-T buffer. IRDye^®^ 680LT Goat anti-Mouse IgG (Li-Cor, Lincoln, NE, USA, cat#926-68020, diluted 1:10,000) was used as the secondary antibody. After incubating with the secondary antibody at room temperature for 1 h in darkness, the membrane was washed 3 times with PBS-T buffer. LI-COR Odyssey infrared imaging system was used for detection.

### 4.6. Cellular Localization Analysis

For cellular localization analysis, the SGCP1 coding region with and without the signal sequence was fused to an mCherry reporter gene creating plasmid constructs pK7WG2D-SGCP-mCherry and pK7WG2D-SGCP∆SP-mCherry, respectively. The two constructs (pK7WG2D-SGCP-mCherry and pK7WG2D-SGCP∆SP-mCherry) were expressed in B301 roots as described above and the presence of the fusion proteins was examined in the transgenic root tissue by using a Leica STELLARIS 8 confocal microscopy system. Visualization of mCherry was performed by excitation at 587 nm and monitoring emission at 610 nm (using a 564–630 nm range filter), and EGFP visualization was performed by excitation at 488 nm and monitoring emission at 509 nm (using a 497–544 range filter).

## Data Availability

The original contributions presented in this study are included in the article. Further inquiries can be directed to the corresponding author.
